# Mos in the Oocyte: How to Use MAPK Independently of Growth Factors and Transcription to Control Meiotic Divisions

**DOI:** 10.1155/2011/350412

**Published:** 2010-12-19

**Authors:** Aude Dupré, Olivier Haccard, Catherine Jessus

**Affiliations:** ^1^CNRS, UMR 7622-Biologie du Développement, 9 Quai Saint-Bernard, 75005 Paris, France; ^2^Université Pierre et Marie Curie-Paris6, UMR 7622-Biologie du Développement, 9 Quai Saint-Bernard, 75005 Paris, France

## Abstract

In many cell types, the mitogen-activated protein kinase (MAPK) also named extracellular signal-regulated kinase (ERK) is activated in response to a variety of extracellular growth factor-receptor interactions and leads to the transcriptional activation of immediate early genes, hereby influencing a number of tissue-specific biological activities, as cell proliferation, survival and differentiation. In one specific cell type however, the female germ cell, MAPK does not follow this canonical scheme. In oocytes, MAPK is activated independently of growth factors and tyrosine kinase receptors, acts independently of transcriptional regulation, plays a crucial role in controlling meiotic divisions, and is under the control of a peculiar upstream regulator, the kinase Mos. Mos was originally identified as the transforming gene of Moloney murine sarcoma virus and its cellular homologue was the first proto-oncogene to be molecularly cloned. What could be the specific roles of Mos that render it necessary for meiosis? Which unique functions could explain the evolutionary cost to have selected one gene to only serve for few hours in one very specific cell type? This review discusses the original features of MAPK activation by Mos and the roles of this module in oocytes.

## 1. Introduction

In many cell types, the mitogen-activated protein kinase (MAP kinase/MAPK) also named extracellular signal-regulated kinase (ERK) is activated in response to a variety of extracellular growth factor-receptor interactions at the cell surface and leads to the transcriptional activation of immediate early genes. Briefly, tyrosine kinase receptors and p21^Ras^ recruit the Ser/Thr kinase Raf-1 that activates MEK (MAP/ERK kinase), which in turn phosphorylates and activates MAPK, hereby influencing a number of tissue-specific biological activities in diverse cell types, as cell proliferation, survival, and differentiation. In one specific cell type, however, the female germ cell, MAPK does not follow this canonical scheme. In oocytes, MAPK is activated independently of growth factors and tyrosine kinase receptors, acts independently of transcriptional regulation, controls the G2-M period of the cell cycle and not the G1-S transition, and is under the control of a peculiar upstream regulator called Mos.

In the early 1980s,* Mos* was originally identified as the transforming gene of Moloney murine sarcoma virus (v-*mos*) causing cellular transformation [1–3]. Its cellular homologue was the first proto-oncogene to be molecularly cloned. When ectopically expressed, the c-*mos* proto-oncogene product Mos induces oncogenic transformation of somatic cells [1]. Mos is a Ser/Thr kinase whose transforming activity strictly depends on its kinase activity. Given this activity as an oncoprotein, its expression pattern was surprisingly restricted to germ cells. In frogs, birds, and mammals, very low concentrations of c-*mos* transcripts were detected in brain and testes, but a high level was observed in ovaries, restricted to oocytes [4–6]. The Mos protein is expressed even in a much more restricted manner than its transcripts, both temporally and spatially at a very specific place: it accumulates during the oocyte meiotic divisions and undergoes selective proteolysis upon fertilization in all eumetazoan except the nematode *Caenorhabditis elegans *where the gene is absent [4, 7–9]. It has to be noted that in mouse and the jellyfish* Clytia hemispherica*, Mos is also expressed in developing spermatids [8–10]. This unique pattern of Mos expression strongly suggested that its function is restricted to meiosis. This hypothesis was confirmed in 1988-1989 by a series of pioneer articles of Noriyuki Sagata, George Vande Woude, and their collaborators who proposed that in the frog oocyte, Mos would be an essential regulator of the universal eukaryotic inducer of M-phase of the cell cycle, MPF (M-phase promoting factor), responsible for reinitiation of meiotic division. MPF is a dimer formed of a catalytic subunit, the Cdk1 kinase (cyclin-dependent kinase 1) and of a regulatory subunit, cyclin B. Mos would serve to activate MPF for entry into the first meiotic division, but also to stabilize MPF during the second meiotic division, inducing the arrest of oocyte that awaits sperm entry [7, 11, 12]. Later, the first function of Mos as an activator of MPF and an initiator of oocyte meiotic entry was not found to be conserved in all animal species whereas its role in postmeiotic arrest turned out to be well conserved: Mos holds in check the unfertilized oocyte arrest. This arrest is characteristic of the entire animal kingdom and is critical for the embryonic development as it allows the mature oocyte to await fertilization, preventing the continuation of cell cycles after meiosis and parthenogenetic development. The target of Mos was discovered few years later. In oocytes, Mos is the upstream activator of MAPK which functions through direct phosphorylation of MEK. Therefore, this original signaling module, Mos/MEK/MAPK, is a critical regulator of some of the most important cell divisions in our life: the meiotic divisions that produce the egg.

At first glance, the physiological meiotic functions of Mos appear to be strikingly different from the oncogenic properties of the kinase in somatic cells. The loss of Mos in mouse leads to parthenogenesis and to the development of ovarian teratomas [13, 14], consistent with the idea that *mos* could be considered as a meiotic tumour-suppressor gene whereas its activity as an oncoprotein is well established in somatic cells. This opens the question of the apparently conflicting effects of the Mos protein, namely, its ability to induce M-phase entry of oocytes, to arrest mitotic cleavage of *Xenopus* unfertilized oocyte and to transform mammalian fibroblasts. Another question arises from the observation that the functions of Mos in the female germ cell appear to be largely mediated by MAPK. In the animal oocytes, Mos is a MAPKKK, equivalent to the proto-oncogene Raf-1. Although Raf-1 is expressed in oocytes, Mos is selected to activate the MAPK module during meiotic divisions. What could be the specific roles of Mos that render it necessary for meiosis and that cannot be played by Raf-1? Which unique functions could explain the evolutionary cost to have selected one gene to only serve for few hours in one very specific cell type? This paper discusses the original features of MAPK activation by Mos and the roles of this module in oocytes.

## 2. Biochemical Properties and Evolution of Mos

In human, mouse, and chicken, the c-*mos* genomic locus contains only a single coding exon corresponding to a poorly conserved *src* homology sequence that yields a protein of 39 kDa. There are two types of conserved regions among Mos proteins: those conserved among all members of the *src* kinase family that are important for maintaining the basic structure for kinase activity and those conserved only among the Mos proteins [6]. The mutations of Mos that cause the loss of its kinase activity (e.g., substituting an arginine for the conserved lysine residue (K90) found in the ATP-binding site) abolish all its biological functions, meiotic induction, meiotic egg arrest, and transforming activity [15, 16]. Several studies have revealed the contribution of some regions of the sequence to Mos functions, as the helix C laying in the kinase domain whose orientation could govern Mos kinase activity [17, 18]. Mutagenic analysis outside the kinase domain showed that the cytoplasmic localization of the protein is important for its biological activity, as the deletion of a 10 amino acids region required for nuclear localization greatly enhances the transforming activity of Mos [19]. Mos also exhibits an *in vitro* DNA-binding activity [20] that seems not to be required for any of its biological functions.

### 2.1. V-Mos versus C-Mos

Mos was originally identified as the transforming gene of Moloney murine sarcoma virus of which several viral isolates have been characterized [1, 3, 21]. Sequence comparison of different v-Mos proteins with murine c-Mos revealed the presence of additional 31 amino acids at the N-terminus of the v-Mos proteins derived from the viral *env* gene and from an upstream extension of the c-*mos* open reading frame. Apart from this N-terminal extension, the amino acid sequences of v-Mos and c-Mos either are identical or differ in few amino acids, depending on the viral strains [3, 18]. *In vivo*, both c-Mos and v-Mos are able to cause oocyte maturation, meiotic oocyte arrest, and transformation of somatic cells [22, 23]. Interestingly, *Mos* genes are able to transform mouse NIH 3T3 cells with markedly different efficiencies depending on the species but not related to the viral origin of the gene: v-Mos and mouse and chicken c-Mos are equally efficient but more than human and *Xenopus* c-Mos [16]. *In vitro*, v-Mos is able to autophosphorylate whereas in parallel experiments c-Mos is not, suggesting a correlation between the transforming activity of v-Mos and its ability to autophosphorylate. However, several point mutations in v-Mos resulted in mutants retaining transforming activity but lacking autophosphorylating activity, showing the functional uncoupling of autophosphorylation and transformation ability [17, 24]. In summary, c-Mos and v-Mos do not exhibit very striking differences in both sequences and cellular activities.

### 2.2. Mos Is a MAPKKK

Given the critical role played by Mos in animal oocyte meiosis and its oncogenic properties, the identification of its targets was of prime importance. In 1993, several laboratories discovered that in *Xenopus* oocytes, oocyte extracts and mammalian cultured cells, either endogenous or exogenous Mos can activate MAPK by directly phosphorylating and activating MEK1, an immediate upstream activator of MAPK [25–28]. The two amino acids phosphorylated by Mos in MEK are identical to those phosphorylated by Raf-1 [29, 30]. Inside the Mos/MEK/MAPK module, both activation reactions (the phosphorylations of MEK and MAPK) require two phosphorylations on the downstream kinase, producing a cascade in which the activity of MAPK varies as a fourth power of the activity of Mos, so that a modest increase in Mos, as doubling its activity, changes the activity of MAPK from 10% to 90%. The Mos/MAPK cascade is, therefore, ultrasensitive, explaining how the activation of MAPK switches from off to on in response to the minute amount of viral Mos protein present in transformed mouse cells (0.0005% of total protein) or in *Xenopus* matured oocytes (200 pg or 0.001% of total protein), accounting for the all-or-none character of this cell fate switch [7, 31]. However, during *Xenopu*s oocyte maturation, progesterone induces the synthesis of Mos before MPF activation whereas MAPK, which preexists in immature oocytes is activated at time of MPF activation, that is one or two hours after Mos synthesis has begun [7, 25, 32]. This observation is difficult to reconcile with the all-or-none and ultrasensitive response of MAPK activation to low amounts of Mos. It is possible that initial synthesized Mos is kept at a too low level to activate the cascade, until its stabilization is achieved by MPF and allows the formation of suprathreshold levels required for turning on the MEK/MAPK module [33].

### 2.3. Other Substrates for Mos?

Other proteins have been proposed to be direct targets for Mos since they can be phosphorylated by Mos *in vitro*. Among them is cyclin B2, the main Cdk1 partner forming the pre-MPF complexes in *Xenopus* immature oocytes. Cyclin B phosphorylation by Mos has been proposed to be necessary for activation of MPF kinase during *Xenopus* oocyte maturation and to prevent degradation of cyclin B during the meiotic arrest [34], a model that has never been experimentally proved. Mos has also been described as a tubulin-associated protein kinase [35–37]. Interestingly, in oocytes from mouse, *Xenopus*, starfish, and the jellyfish *Clytia hemispherica*, Mos activity is required for the correct formation and peripheral positioning of the meiotic spindle [8, 9, 38, 39]. Mos is also localized on mitotic spindle and spindle pole regions in Mos-transformed NIH/3T3 cells [35, 36]. The tubulin kinase activity of Mos could thus participate in the modification of microtubules and contribute to the formation of the spindle. However, it is not known whether these *in vitro* substrates do mediate the physiological functions of Mos. It has also been proposed that in mouse oocytes, Mos would contribute to MAPK activation not only through MEK activation but also through the inhibition of an unidentified phosphatase [40], an interesting observation that has not been noted in other species until now.

### 2.4. Mos Evolution

For more than 20 years, the studies on Mos have been conducted in vertebrates, mainly mouse, human, birds, and frogs. In the entire animal kingdom, to maintain ploidy through successive generations, meiosis must be followed by mitosis after the recovery of diploidy by fertilization. The coordination from meiotic to mitotic cycle is ensured by a meiotic arrest of the oocyte, while the cell awaits fertilization. This arrest occurs at metaphase of the second meiotic division (metaphase II) in vertebrates whereas the stage of oocyte meiotic arrest is variable in invertebrates. It was clearly established in vertebrates that Mos is essential to arrest oocyte meiotic divisions at metaphase II, leading to the hypothesis that Mos is a molecular regulator of MPF and the M-phase of the cell cycle. For this reason, it was assumed that Mos functions only in vertebrate oocytes, until Tachibana and his collaborators isolated the first invertebrate Mos from starfish and demonstrated that it is essential for the natural arrest of the echinoderm unfertilized egg, in G1 phase after completion of meiotic divisions [8]. These results changed the view on the role of Mos. It is not restricted to the maintenance of the metaphase II arrest but more broadly prevents the meiotic/mitotic conversion by arresting the unfertilized oocyte at various stages of meiosis depending on species. The proposal of a conserved role of Mos in invertebrate and vertebrate oocytes was then questioned by the observation that the *mos* gene is absent in the nematode *Caenorhabditis elegans* genome and that the *Drosophila* Mos ortholog is not essential for meiosis [41]. However, in the sawfly, where Mos has also been characterized, it mediates the physiological metaphase I arrest characterizing the insect oocytes [42]. A recent phylogenetic survey reconciled these data by demonstrating that Mos appeared early during animal evolution as an oocyte-expressed kinase and functioned ancestrally in regulating core specializations of female meiosis [9]. Unexpectedly, cnidarian genomes contain more than one *mos* gene after ancestral duplications, in contrast with bilaterians, in which only one single gene is found. Changes in the characteristics of oogenesis probably explain this diversification of *mos* genes and their related functions in cnidarians. All genomes from eumetazoan (bilaterian + ctenophore + cnidarian) species examined by Amiel et al. [9] contained *mos* gene orthologs, showing well-conserved kinase domains, with the exception of *C. elegans* where the gene was secondarily lost. *Mos* genes were not detected in available sponge (*Amphimedon*), choanoflagellate, or fungi genomes, suggesting that the gene may have originated in a common eumetazoan ancestor [9]. Thus, Mos kinases should be seen not as core regulators of meiosis, which is a much older process than Mos origin, but of a particularity of meiosis in eumetazoan. Which innovations of oocyte meiotic divisions relate to *mos *gene evolution have to be investigated.

## 3. Oocyte Meiotic Maturation: The Unique Physiological Process under Mos Control

**Figure 1:**
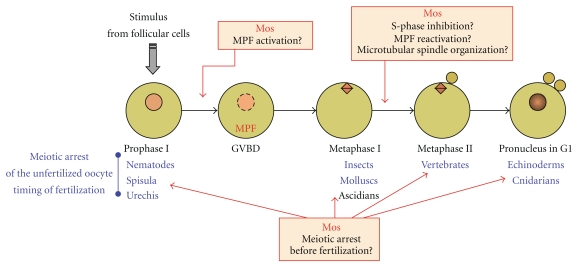
Progression through meiosis and timing of fertilization: what does Mos do? Oocytes are arrested at prophase of the first meiotic division (prophase I). Under response to a physiological stimulus, MPF is activated and promotes breakdown of the nuclear envelope (GVBD for germinal vesicle breakdown) and formation of the metaphase I spindle. In insects, molluscs, and ascidians, oocytes arrest at metaphase I until fertilization. In the other cases, oocytes extrude the first polar body and enter the second meiotic division. In vertebrates, they arrest at metaphase II until fertilization. In echinoderms and cnidarians, they complete the second meiotic division by emitting the second polar boy, reform a nucleus (female pronucleus), and stop at the G1 phase of the first cell cycle until fertilization. In different species including the nematode *Caenorhabditis elegans*, fertilization occurs at prophase I and corresponds to the stimulus promoting meiotic maturation. Mos has been implicated: (i) in the initial step of MPF activation during reinitiation of meiotic division, (ii) during the metaphase I to metaphase II transition for the suppression of S-phase, for the microtubular spindle organization and for the reactivation of MPF to enter meiosis II, and (iii) in the arrest of oocyte maturation before fertilization.

**Figure 2:**
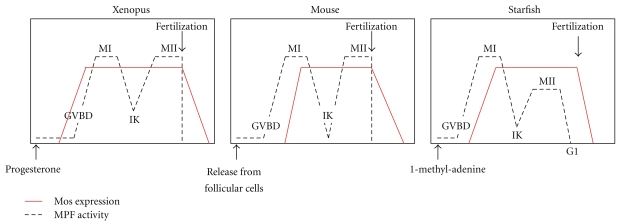
Patterns of Mos expression and MPF activity during oocyte meiotic maturation and at fertilization in *Xenopus*, mouse, and starfish. Prophase I-arrested oocytes require a physiological stimulus to undergo meiotic maturation: progesterone in frogs, release from the follicle in mammals, and 1-methyl-adenine in starfish. Once activated, MPF promotes entry into the first meiotic division: breakdown of the nuclear envelope (GVBD for germinal vesicle breakdown) and formation of the metaphase I spindle (MI). MPF activity falls due to partial cyclin degradation at meiosis I/meiosis II transition or interkinesis (IK), during which chromosomes remain condensed without nuclear membranes and in the absence of DNA replication. MPF rises again leading to entry into meiosis II. In vertebrates, oocytes arrest at metaphase II (MII), while in echinoderms, oocytes complete the second meiotic division and arrest at the G1 phase. Mos translational timing is different among species, occurring before MPF activation in *Xenopus* (however, Mos protein is unstable until GVBD and MAPK activity is detected only at time of MPF activation, not illustrated) and during metaphase I in other species.

In the animal kingdom, the oocytes growing in the ovaries are arrested at prophase of the first meiotic division that resembles a G2-phase of the cell cycle. These immature oocytes require a physiological stimulus to undergo meiotic maturation: the progression through the meiotic divisions that converts them into fertilizable oocytes, again arrested at various stages of meiosis and awaiting fertilization (Figure 1). Indeed, the embryonic development cannot begin until completion of the female germ cell meiotic divisions. This temporal coupling is ensured by the arrest of meiotic divisions of the oocyte that depends on a biological activity called CSF (for cytostatic factor) [43, 44]. The CSF arrest is released by fertilization. Oocytes arrest at metaphase I in insects, molluscs and ascidians and at metaphase II in vertebrates. In echinoderms and cnidarians, oocytes complete meiosis and arrest in G1 (and are then called “eggs” in these species, as they completed meiotic divisions). In different species including the nematode *Caenorhabditis elegans*, fertilization occurs at prophase I and corresponds to the stimulus promoting meiotic maturation. Except for this last case, the stimuli for maturation are provided at ovulation by the follicular cells surrounding the oocyte. The signals are very different from species to species: steroid hormones in frogs and fishes, modified purines in starfish, removal of a follicular inhibitor in mammals, but all activate signaling pathways that converge to the same target, independently of transcription: the activation of the universal eukaryotic inducer of M-phase, MPF, a complex formed of the Cdk1 kinase, and Cyclin B. Once activated, MPF promotes entry into the first meiotic division: breakdown of the nuclear envelope (known as GVBD for germinal vesicle breakdown) and formation of the metaphase I spindle (Figures 1 and 2). MPF activity falls during anaphase I, due to partial cyclin B degradation, and rises again leading to entry into meiosis II. Importantly for the generation of proper haploid gametes, DNA replication does not occur between both meiotic divisions. The need for the Mos/MAPK cascade during oocyte meiotic maturation has been debated for decades (Figure 1). First, Mos has often been implicated in the initial step of MPF activation during reinitiation of meiotic division, especially in the frog oocyte. Second, Mos has been shown to be required during the metaphase I to metaphase II transition for the suppression of S-phase and for the reactivation of MPF after meiosis I, thus enabling the oocyte to enter meiosis II. Third, a universal role of Mos is to prevent parthenogenesis by arresting oocyte maturation at the various stages depicted in Figure 1, allowing them to await fertilization. Mos is therefore a key regulator of meiosis in the animal kingdom.

## 4. Regulation of Mos Activity

Mos protein functions as a kinase in a meiosis-specific manner in animal oocytes. In *Xenopus*, *Mos* gene is actively transcribed in the grownup oocytes where its message is abundant [6]. However, oocytes arrested at prophase I lack detectable levels of Mos. The synthesis of the protein is induced in response to the physiological stimulus that promotes reentry into meiotic divisions, Mos protein then accumulates throughout meiotic maturation, is stably maintained in the mature oocyte, and is finally degraded at fertilization (Figure 2). This unique pattern of Mos expression, accounting for its restricted function during oocyte meiosis, is clearly under a tight translational and proteolysis control.

### 4.1. Translational Control of Mos

Many studies have been devoted to the regulation of translation of maternally stored mRNAs during meiosis resumption of *Xenopus* oocytes. In prophase-arrested oocytes, translation is repressed. Progesterone, the physiological inducer of meiotic divisions in *Xenopus*, induces the ordered translation of mRNAs based on polyadenylation events. This translational regulation depends on regulatory elements within the 5′ and 3′ untranslated regions (UTRs) of target mRNAs which are recognized by sequence-specific RNA protein complexes to mediate translational control [45, 46].

In immature *Xenopus* oocytes, the kinase TOR (target of rapamycin) controls the translation of RNAs through a 5′TOP (terminal oligopyrimidine) sequence that contributes to suppress translation of other RNAs, including Mos mRNA, until hormonal stimulation of maturation [47]. The translational induction of the dormant mRNA encoding Mos occurs 2 to 3 hours after stimulation by progesterone, before MPF activation [7, 12] (Figure 2). The 3′end polyadenylation of Mos mRNA and a 5′end modification, cap-specific 2′-O-methylation, were shown to be pivotal regulatory steps for translational recruitment and for the progression of *Xenopus* oocytes through meiosis [48, 49]. Cytoplasmic polyadenylation requires two sequences in the 3′UTR of Mos mRNA, the U-rich cytoplasmic polyadenylation element (CPE) and the near-ubiquitous polyadenylation hexanucleotide AAUAAA recognized by the multifactor complex CPSF (cleavage and polyadenylation specificity factor). CPE is recognized by a group of factors among them the two most important are CPEB, the CPE-binding factor, and Maskin [50–52]. In oocytes, Maskin also binds eukaryotic translation initiation factor 4E (eIF4E), an interaction that excludes eIF4G and prevents formation of the eIF4F initiation complex [51]. It has been proposed in *Xenopus* that an early site-specific phosphorylation of CPEB, possibly catalyzed by the Aurora-A kinase [53], would be essential for the polyadenylation of Mos mRNA by the poly(A)polymerase. Once cytoplasmic polyadenylation has been promoted by CPEB, the newly elongated poly(A) tail becomes bound by poly(A)-binding protein (PABP), which in turn binds eIF4G and helps to displace Maskin from eIF4E, thereby inducing translation [51, 52]. Polyadenylation requires two factors, symplekin, a CPEB- and CPSF-binding protein that serves as a scaffold upon which regulatory factors are assembled, and xGLD-2, an unusual poly(A) polymerase that is anchored to CPEB and CPSF even before polyadenylation begins [54].

However, several findings refute the hypothesis that CPE sequences and CPEB alone could account for the range of temporal inductions of maternal mRNAs, including Mos mRNA, observed during *Xenopus* oocyte maturation [55]. Polyadenylation and mRNA translational activation of Mos are also controlled by a distinct CPE-independent mechanism that depends on a 3′UTR polyadenylation response element (PRE) [55, 56]. This translational activation is mediated by the transacting factor Musashi that binds to the PRE of *mos* mRNA [56, 57]. As CPEB, Musashi would also be essential for Mos translational activation during *Xenopus *oocyte meiotic maturation [58]. Several reports suggest that mRNA translation directed by CPE is a late event that would require early Musashi-dependent mRNA translation, implying that Musashi function is necessary to establish the temporal order of maternal mRNA translation during meiotic progression [57–59]. It is, therefore, still difficult to get a clear picture of the signaling events that trigger Mos mRNA polyadenylation and translation in the *Xenopus* oocyte.

Interestingly, MAPK can stimulate Mos synthesis in *Xenopus* oocyte, creating a positive feedback loop. Microinjection of activated forms of MEK or MAPK is sufficient to stimulate Mos mRNA polyadenylation and translation [60, 61] whereas inhibition of MAPK activation prevents Mos accumulation [62]. MAPK activity could contribute to CPEB phosphorylation and activation [63], but the precise mechanism allowing this kinase to stimulate the polyadenylation of Mos mRNA is still elusive.

In starfish, rat, and mouse, the synthesis of Mos is also initiated in maturing oocytes, except that it accumulates only after MPF activation and the first meiotic reentry [8, 64, 65] (Figure 2). This noticeable difference in the translational timing of Mos mRNA in mouse and starfish oocytes compared to *Xenopus* oocyte, explains why Mos is not involved in the activation of MPF and the entry into the first meiotic division in these species [8, 13, 14, 38], a not-so-surprising result given that this process does not depend on protein synthesis. Similarly to the translational regulation described in the *Xenopus* oocyte, mouse Mos mRNA is under the translational control of cytoplasmic polyadenylation, a necessary event for the oocyte progression to meiosis II after the first polar body emission. Cytoplasmic polyadenylation of Mos mRNA in murine oocyte requires three cis elements in the 3′ UTR, the polyadenylation hexanucleotide AAUAAA, and two CPEs [66]. The biochemical events that govern polyadenylation in mouse oocytes are not well known, but they also involve CPEB whose activity is controlled by Aurora-catalyzed phosphorylation, similarly to the *Xenopus* situation [67–69].

Interestingly, one of the two Mos paralogs of the jellyfish *Clytia hemispherica* is subject to differential translational regulation, being expressed during the growth period of oogenesis, before meiotic maturation, perhaps under the control of the TOR pathway [9]. This raises the attractive possibility that Mos may have acquired new roles during evolution after sequence changes in the UTRs affecting translational timing [70, 71]. Acquisition of a 5′TOP sequence in one *Clytia* paralog may have resulted in an earlier translational timing of this paralog compared to the other one, leading to functions during the oocyte growth period preceding meiotic maturation. In *Xenopus*, 3′UTR changes, such as an early acting Musashi PRE, could have resulted in the temporal advancement of Mos translation before MPF activation, explaining its atypical participation in MPF activation and initiation of oocyte maturation. In contrast, the translational activation of the human Mos 3′ UTR is uniquely dependent on a late acting CPE-dependent process [59]. Mos 3′UTR regulatory differences, therefore, underlie species-specific temporal patterns of Mos mRNA translational recruitment during oocyte maturation and hence different temporal windows for its functions, offering it the possibility to regulate MPF activation, or not.

### 4.2. Control of Mos Stability

Even though the synthesis of Mos begins soon after progesterone stimulation in *Xenopus* oocytes, the protein remains unstable and unable to activate MAPK until MPF activation [33, 72, 73]. The polyadenylation-controlled translation of Mos is an early event but is not sufficient for Mos to accumulate. Several studies attempted to elucidate the molecular mechanisms that govern the metabolic stability of Mos during meiotic maturation. Using a number of Mos mutants expressed in *Xenopus* oocytes, Nishizawa et al. [33, 74] demonstrated that the instability of Mos depends on the ubiquitination of Lys34 and is determined primarily by its penultimate N-terminal residue, a proline, and the phosphorylation status of the adjacent serine (Ser3) residue. Clearly, Mos is stabilized by phosphorylation at Ser3, its major phosphorylation site *in vivo* [17]. This critical phosphorylation for Mos stability is catalyzed by MPF kinase activity, which probably acts to prevent the N-terminus of Mos from being recognized by its ubiquitin ligase [33, 74, 75]. Similarly in mouse, the phosphorylation of Ser16 prevents Mos degradation and stabilizes the protein [76]. The unique role of Mos phosphorylation would be to stabilize the protein. Therefore, while the Mos/MAPK pathway can facilitate the activation of MPF, MPF is required to directly phosphorylate and stabilize Mos protein, a mechanism creating a positive feedback loop between Mos and MPF. Importantly, these results reconcile some apparent conflicting conclusions on Mos functions in *Xenopus* and mouse. In both cases, Mos activity can take place only after MPF activation and meiosis reentry: the protein needs to be stabilized by MPF despite an early translational initiation in the frog oocyte, while it is only synthesized after MPF activation in the mouse oocyte.

### 4.3. Control of Mos Degradation at Fertilization

In all species studied until now, fertilization induces a rapid escalation in intracellular calcium ion concentration that releases the meiotic arrest. Interestingly, Mos was proposed to be the cytostatic factor responsible for meiotic arrest of the unfertilized oocytes [12] and was shown to undergo specific proteolysis upon fertilization when the arrest is relieved [7, 77]. However, in both *Xenopus* and mouse oocytes, Mos disappearance at fertilization starts 30 to 45 minutes after the calcium surge [7, 77, 78], whereas MPF inactivation occurs within 15 minutes through cyclin B degradation (Figure 2). Clearly, the degradation of Mos at fertilization is not required for cyclin B proteolysis and is, therefore, not the inducer of the release of the meiotic arrest but rather a consequence of this exit [79]. To summarize, Mos is essential for establishing and maintaining the meiotic arrest; the release of this arrest at fertilization is due to a mechanism that overcomes Mos but is not a consequence of Mos degradation. In *Xenopus* oocytes, MPF ensures Mos stability by phosphorylating its Ser3 residue. At fertilization, cyclin B is rapidly degraded, leading to the inactivation of MPF and consequently the dephosphorylation of Mos. Under this unphosphorylated state, Mos is then degraded by a yet unidentified ubiquitin ligase, different from the anaphase-promoting complex-cyclosome (APC/C) that targets several cell-cycle regulatory proteins, including cyclin B, for destruction [33, 74, 75]. However, the destruction of Mos under the control of MPF appears to be specific to vertebrate oocytes that are arrested in M-phase. It would be important to evaluate how unfertilized eggs of invertebrates, arrested after meiosis completion in G1, get rid of Mos, an essential event to prevent the organism from the oncogenic activity of this protein.

## 5. Which Functions for Mos in Oocytes?

### 5.1. Mos as an Initiator for Oocyte Maturation?

The very first studies on the physiological function of Mos were conducted on the *Xenopus* oocytes and revealed that injection of Mos antisense oligonucleotides blocks GVBD and MPF activation whereas the injection of Mos RNA activated MPF and induced GVBD in the absence of progesterone [6, 11]. In contrast to mouse or starfish, a period of protein synthesis is necessary for MPF activation in the frog oocyte [80]. Yew et al. [81] reported that Mos protein efficiently induces GVBD and the activation of MPF in the absence of protein synthesis (but in the presence of low concentrations of progesterone unable to trigger meiotic maturation), leading to the conclusion that Mos is the only synthesized protein required for initiating maturation. All constitutively active downstream effectors of Mos, MEK, MAPK, and p90^Rsk^, are also able to induce meiotic maturation when microinjected into oocytes [82–84]. The effects of Mos on MPF activation are mediated through MEK/MAPK/p90^Rsk^, as microinjected Mos is inactive in the presence of the pharmacological MEK inhibitor, U0126 [85]. Altogether, these results led to a simple scenario, where MPF activation is the result of a linear chain of molecular events initiated by progesterone, starting with the synthesis of Mos protein, the subsequent activation of the MEK/MAPK/p90^Rsk^ cascade that would eventually control the two regulators of the Cdk1 catalytic subunit of MPF, the Myt1 kinase that phosphorylates and inactivates Cdk1 and the Cdc25 phosphatase that specifically activates Cdk1 [86–88].

However, this simple view was then questioned by several studies. Gross et al. and Fisher et al. showed that progesterone is able to activate MPF by a mechanism independent of MAPK [85, 89]. This conclusion is difficult to reconcile with a requirement for Mos downstream of progesterone in *Xenopus* oocyte. Consistent with the idea that MAPK activation downstream of Mos synthesis is not required for maturation, inhibition of Mos synthesis by morpholino antisense oligonucleotides fails to block progesterone-stimulated GVBD [90]. This conflicting results on the requirement of the Mos/MAPK cascade to activate MPF in *Xenopus* oocyte were recently reconciled. It was shown that MPF activation induced by progesterone is completely abolished when cyclin B synthesis and the Mos/MAPK pathway are simultaneously impaired [88]. The replenishment of at least one of these pathways restores MPF activation. Altogether, these results demonstrate that MPF activation requires either the Mos/MAPK pathway or cyclin B synthesis. Each of these pathways can bypass the deficiency of the other one. In contrast to cyclin B1 accumulation induced by progesterone independently of MPF activation, the strong accumulation of Mos requires a stabilizing phosphorylation catalyzed by MPF [73, 75] and as a consequence MAPK activation only takes place when MPF activation is already initiated. This differential regulation in the accumulation of Mos and cyclin B1 suggests that the physiological pathway induced by progesterone depends on cyclin B synthesis and that the Mos/MAPK cascade contributes to MPF activation once Mos stabilization is achieved. When cyclin B synthesis is impaired, some rescue mechanism could recruit the Mos/MAPK pathway, allowing it to complement the deficiency in cyclin B synthesis. These findings solve the paradoxical situation of the frog oocyte meiotic reentry control, where the Mos/MAPK activation was considered as necessary for MPF activation, while this is clearly not the case in all other systems studied until now (mouse, starfish, jellyfish, *Drosophila,* and *C. elegans, *where Mos is not expressed yet at the time of MPF activation, Figure 2) and integrates the *Xenopus* in the context of a universal mechanism for meiotic maturation in oocytes throughout the animal kingdom.

### 5.2. Suppression of DNA Replication and Entry into Meiosis II

In *Xenopus*, the ability to replicate DNA is acquired during maturation at the beginning of meiosis I by synthesis of the only missing replication factor, Cdc6, which is essential for recruiting the minichromosome maintenance (MCM) helicase to the prereplication complex [91, 92]. After GVBD, the maturing oocyte is thus fully equipped with a functional replication machinery that has to be inhibited to prevent the entry into S-phase until fertilization. In *Xenopus,* Mos has been shown to be required during the metaphase I to metaphase II transition to suppress S-phase. When the synthesis or the activity of Mos is specifically inhibited or when MAPK activation is prevented by U0126, *Xenopus* oocytes complete meiosis I but a nuclear envelope reforms and DNA replication occurs [85, 90, 93]. Essentially similar results have been obtained in starfish oocytes [8]. However, in mouse oocytes, conflicting results have been obtained concerning the involvement of Mos and MAPK in S-phase suppression and entry into meiosis II. The ablation of Mos by antisense oligonucleotides either arrests oocytes before extrusion of the first polar body [4] or induces nuclear reformation and DNA replication after meiosis I [94, 95], as in *Xenopus*. In contrast, oocytes from the *mos* gene knockout mice enter meiosis II essentially normally, despite going through an interphase-like microtubular stage [13, 14, 38]. The reasons for these conflicts could be due to differences in the strains of mice used or to the experimental strategy used for deletion. In jellyfish oocytes where Mos is ablated by morpholino antisense or MAPK activation is inhibited by U0126, GVBD occurs on time, but oocytes show a complete failure to emit both first and second polar bodies. However, they do not reform a replicating nucleus [9]. Then, the ability of Mos to suppress DNA replication between the two meiotic divisions of the oocyte is not a function universally conserved. These differences could be related to the presence or the absence of a functional replicative machinery in oocytes, depending on the species. For instance, mouse oocytes do not acquire the ability to replicate DNA before metaphase II (and therefore do not need to suppress it), in contrast to starfish and *Xenopus* oocytes that develop the ability to replicate DNA early after GVBD [8, 91–93].

Until now, the molecular mechanism controlled by the Mos/MAPK cascade and leading to the inhibition of DNA replication remains unclear. All the findings support the view of a critical function of Mos at meiosis I-meiosis II transition in vertebrates: the Mos/MAPK module is involved in MPF reactivation that depends both on the arrest of cyclin B degradation, initiated at the exit of meiosis I, and on new cyclin B synthesis, allowing MPF reactivation and entry into meiosis II [85, 90, 93, 96]. By controlling this cyclin B turn-over, Mos allows MPF activation and entry into meiosis II. The Mos/MAPK module could act indirectly on the replication machinery through the control of MPF activity. Since quite similar failure of the meiosis I/meiosis II transition occurred in *Xenopus* oocytes that were injected either by antisense against Mos mRNA or dominant-negative Cdk1 kinase [93], it was suggested that MPF reactivation occurring under the control of Mos/MAPK after meiosis I would suppress DNA replication. However, when the reactivation of MPF at meiosis II is inhibited specifically by antisense oligonucleotides against B-cyclins, the *Xenopus* oocytes degenerate, fail to form a second meiotic spindle, but do not support nuclear organization and DNA replication [96]. This careful analysis favors the view that the Mos/MAPK pathway acts directly to suppress DNA replication, independently of MPF activity.

A conserved function for Mos revealed by observations in the oocytes of starfish, mouse, *Xenopus,* and the jellyfish *Clytia* is its involvement in the control of spindle formation and positioning and the chromatin organization. This was first revealed by analysis of mouse oocytes [38, 97, 98]. Remarkably in *mos* ^−/−^ oocytes or in oocytes where MEK is inhibited, the microtubules and chromosomes evolve towards an interphase-like state during the transition between two meiotic M-phases and then exhibit monopolar half-spindles [38, 97–99]. Later on, similar observations were performed in other systems [8, 9, 39]. This ancient and conserved role of the Mos/MAPK in the modulation of microtubular cytoskeleton to assure meiotic spindle formation and positioning could contribute to its cytostatic activity independently on the control of MPF in oocytes arrested at metaphase I (as *Drosophila*) or metaphase II (as in mouse, see next paragraph). It could also contribute to the chromosome instability of tumor cells where *mos* is upregulated [100].

### 5.3. The Meiotic Arrest of the Unfertilized Oocyte: A Conserved Function of Mos

In all animals, oocytes halt meiosis to prevent embryonic development in the absence of fertilization. The stage of meiosis at which the oocyte (also called “egg” in species where meiotic divisions are completed, as echinoderms and cnidarians) typically arrests varies depending species: metaphase II in vertebrates, metaphase I in insects, molluscs, and ascidians, G1 phase following meiosis in echinoderms and cnidarians, prophase I in nematodes (Figure 1). Surprisingly, given the diversity of stages where the cell cycle is halted, Mos was found to be the ubiquitous cytostatic factor responsible for the unfertilized oocyte arrest throughout the animal kingdom.

#### 5.3.1. Metaphase II Arrest in Vertebrates

The activity responsible for this arrest was first described in 1971, by injecting extracts derived from metaphase II-arrested oocytes into one blastomere of a 2 cell-stage *Xenopus* embryo [12, 43]. The injected blastomere arrests at the next mitosis with a metaphase spindle and a high MPF activity. On the basis of these observations, Masui and Markert established the existence of an activity called CSF (cytostatic factor) that is responsible for metaphase II-arrest. Using this assay, Sagata et al. [12] proposed that Mos is a cytostatic factor responsible for meiotic arrest in vertebrate eggs. Since then, the downstream targets of Mos, MEK, MAPK, and p90^Rsk^ were also shown to cause an M-phase arrest when injected in a *Xenopus* dividing blastomere [101–104]. The ability of Mos to confer a CSF arrest is abrogated when MAPK activity is prevented [101, 104, 105]. Conversely, any upstream activator of MAPK is able to induce a CSF arrest, as the small G protein Ras or the kinase Raf [106, 107]. These results suggest that the metaphase II-arresting CSF activity of Mos is largely mediated by MAPK. Nevertheless, the physiological validation of the Mos/MAPK pathway as CSF came from the deletion of Mos in oocytes. Ablating Mos synthesis by an antisense strategy in *Xenopus* oocytes induces cell cycle oscillations after meiosis I, mimicking the early embryonic cell cycles [90]. Invalidating the *mos* encoding gene in mouse leads to a failure of mature oocytes to arrest during meiosis and as a consequence to parthenogenetic activation of unfertilized oocytes [13, 14, 38].

**Figure 3:**
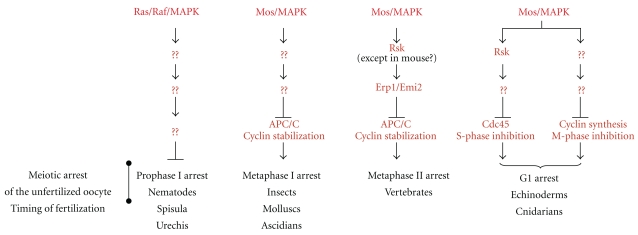
Meiotic arrest of the unfertilized oocyte: the downstream effectors of Mos/MAPK. In all species, oocytes halt meiosis to prevent embryonic development in the absence of fertilization. Depending on species, meiosis arrests at prophase I, metaphase I, metaphase II, or G1 following meiosis. Except in *C. elegans*, Mos was found to be the ubiquitous cytostatic factor responsible for the unfertilized oocyte arrest. Its downstream targets accounting for the meiotic arrest of the unfertilized oocytes are indicated.

In vertebrates, the Mos/MAPK pathway serves to stabilize MPF, ensuring an arrest at the metaphase stage. Releasing this blockage requires the activity of the APC/C protein complex, a ubiquitin ligase that targets cyclin B for destruction [108, 109]. In mouse and *Xenopus* metaphase II-arrested oocytes, APC/C is directly inhibited by the Erp1/Emi2 protein [110]. During oocyte maturation, Erp1/Emi2 appears only after metaphase I, explaining why the oocyte does not stop at metaphase I, but at metaphase II [111, 112]. Upon fertilization, a transient rise in free intracellular calcium activates calmodulin-dependent protein kinase II that phosphorylates Erp1/Emi2, thereby creating a docking site for the Polo kinase. The Erp1/Emi2 phosphorylation by Polo kinase targets it to destruction, releasing APC/C from inhibition [110, 113, 114]. As a consequence, cyclin B is degraded, MPF activity is thus inactivated and the fertilized oocyte exits metaphase II. Given that Erp1/Emi2 can itself inhibit APC/C and stabilize MPF, why is the Mos/MEK/MAPK/p90^Rsk^ required for metaphase II arrest? Recent works provided promising clues about the relationships between Mos and Erp1/Emi2. In *Xenopus* metaphase II-arrested oocytes, Erp1/Emi2 is a substrate of p90^Rsk^, and Mos-dependent phosphorylation of Erp1/Emi2 by p90^Rsk^ is crucial for both stabilizing Erp1/Emi2 and establishing CSF arrest in meiosis II oocytes [115, 116]. More precisely, the Rsk-mediated phosphorylation of Erp1/Emi2 promotes its interaction with the protein phosphatase PP2A. PP2A dephosphorylates two distinct clusters of residues in Erp1/Emi2, one responsible for modulating its stability during the metaphase II-arrest and one controlling its binding to the APC/C [112, 117, 118]. Thus, Mos and Erp1/Emi2 collaboratively establish and maintain metaphase II arrest in *Xenopus* oocytes (Figure 3).

In mouse, the APC/C Erp1/Emi2 also plays a critical role for maintaining the metaphase II-arrest of the unfertilized oocyte [119, 120]. Therefore, it would be logical to predict that the mechanism of metaphase II-arrest establishment is also conserved between frog and mouse. However, this is not so. Surprisingly, although it is well established that the Mos and MAPK are essential for establishing this arrest, p90^Rsk^, which is activated by MAPK as in the other species [121], is not involved in the metaphase II-arrest of mouse oocytes [122]. This is in strong contrast with *Xenopus* or starfish unfertilized oocytes, where it is the main mediator of Mos cytostatic activity (see before and below). Then, the downstream effector of MAPK regulating APC/C through Erp1/Emi2 and/or stabilizing the microtubular spindle still requires to be elucidated in mouse.

#### 5.3.2. G1 Arrest in Starfish and Jellyfish

In contrast to vertebrates whose oocytes arrest at metaphase II to await fertilization, unfertilized eggs of starfish are blocked at G1 phase after completion of meiosis II. In the absence of Mos, meiosis I is directly followed by repeated embryonic mitotic cycles in starfish [8]. Therefore, despite the difference in the arrest stage between vertebrates and starfish, both arrests depend on the same Mos-MAPK pathway, indicating that the difference relies on particular downstream effectors. Inhibition of p90^Rsk^ activity in the starfish unfertilized egg leads to S-phase in the absence of fertilization [123], indicating that the effects of Mos on the meiosis arrest are mediated, at least partially, through its downstream effector, p90^Rsk^, as in *Xenopus* oocyte. In starfish G1-arrested eggs, chromatin is loaded with the MCM complex to form the prereplicative complex. p90^Rsk^ blocks S-phase entry by preventing the further loading of Cdc45 onto chromatin to form the preinitiation complex and the subsequent initiation of DNA replication [124] (Figure 3). However, the S-phase induced by p90^Rsk^ inhibition is not followed by M-phase when MAPK remains active, owing to repression of cyclin A and B synthesis (Figure 3). By contrast, inactivation of MAPK alone induces M-phase. Thus, there is a divergence of separate pathways downstream of MAPK that together block the initiation of the embryonic mitotic cycle. One depends on p90^Rsk^ and prevents S-phase, the other is not mediated by p90^Rsk^ and leads to the prevention of the first mitotic M-phase through suppression of mitotic cyclin synthesis (Figure 3). Release from this dual lock by fertilization results in the start of the embryonic cell cycle [125].

In the jellyfish *Clytia hemispherica* as in starfish, unfertilized eggs are blocked in G1 phase after meiotic division completion and the invalidation of the Mos/MAPK pathway leads to parthenogenetic development with complete cleavages, revealing that the Mos/MAPK function in meiotic arrest is conserved through the animal kingdom [9].

#### 5.3.3. Metaphase I Arrest

In many invertebrates, as insects, molluscs, and ascidians, oocytes arrest at metaphase I. At first glance, it is tempting to speculate that the molecular basis of this arrest could resemble those operating during the metaphase II-arrest in vertebrates oocytes, based on the Mos/MAPK pathway as an upstream regulator of cyclin B stabilization through APC/C inhibition. This is not so. The *Drosophila* homolog of Mos has been identified and is expressed specifically in oocytes. As in vertebrates, Mos is responsible for the majority of MAPK activation that occurs during meiotic maturation. Unexpectedly, the oocytes with a Mos deletion complete meiosis normally and produce fertilized embryos that develop [41]. Therefore, the *Drosophila *Mos ortholog would not be essential for meiosis. Some innovation in oocyte function could explain the relaxation of the cytostatic role of Mos in *Drosophila*, as the adoption of internal fertilization that could reduce the delay between meiotic completion and fertilization. It has also to be noticed that the mechanism by which the metaphase I arrest is maintained and released is particularly intriguing in *Drosophila*. It has been demonstrated that chiasmata are essential for signaling the arrest [126, 127], but the role of MPF remains unknown. Then, the molecular mechanisms underlying the metaphase I arrest could regulate very different targets from those identified in other species. In the parthenogenetic insect *Athalia rosae*, the Mos/MAPK module arrests the unfertilized oocytes at metaphase I [128]. Therefore, the role of Mos in *Drosophila *oocyte cannot be extrapolated to all insects (Figure 3).

The oocytes of the ascidian *Ciona intestinalis* merge as a new model to study the meiotic divisions. The Mos/MAP kinase pathway is perfectly conserved in ascidians and metaphase I-arrested oocytes of *C. intestinalis *contain a CSF-like activity able to block cell division in two-cell embryos [129]. MAPK might ensure this activity [130], suggesting that meiotic arrest in *C. intestinalis* could resemble that of vertebrates, such as *Xenopus*, accordingly to the position of this organism in the evolutionary tree (Figure 3).

#### 5.3.4. *C. elegans*: Meiotic Arrest without Mos

The* Caenorhabditis elegans* nematode has secondarily lost the *mos* gene. In this worm, MAPK is activated at two separate steps during the oocyte meiotic cell cycle, under the control of the small G protein Ras and the kinase Raf that replaces Mos [131, 132]. It is first stimulated in the pachytene stage of meiotic prophase I, where it is required for the progression through pachytene. MAPK is then inactivated rapidly after pachytene and remains inactive throughout diakinesis, which is the point of prophase arrest in *C. elegans* oocyte. The prophase arrest is relieved by a maturation signal produced by the sperm that leads to MAPK reactivation [133]. Interestingly, maintaining MAPK in an inactive state after pachytene exit is necessary to allow the developing oocytes to arrest the cell cycle in diakinesis until maturation is induced by the sperm signal. Oocytes with a constitutive MAPK activity after pachytene completion are unable to arrest in diakinesis for a prolonged time, and they enter a mitotic cell cycle without being fertilized [134]. Therefore, despite the lack of *mos* gene in *C. elegans*, MAPK exerts a function during meiosis, being required in oocytes to coordinate meiosis progression with fertilization. However, instead of playing the usual cytostatic role, active MAPK is necessary to break the meiotic arrest where the *C. elegans* oocyte is awaiting fertilization (Figure 3). This observation is not so surprising given the fact that the arrest where the *C. elegans* oocyte awaits fertilization corresponds to the universal arrest, where oocytes await the meiotic maturation signal, prophase I, characterized by the absence of Mos expression through all the animal kingdom.

## 6. Concluding Remarks

Since the discovery of the meiotic functions of Mos about 20 years ago, there has been many studies exemplifying the apparently conflicting effects of the Mos protein, namely, its ability to induce M-phase entry of oocytes, its ability to arrest the meiotic cell cycle at various stages, and its ability to transform mammalian fibroblasts. As reviewed here, these studies can be easily reconciled when taking into account three characteristics of Mos that distinguish it from the main somatic regulators of MAPK, as Raf-1.

First, Mos activates MAPK independently of the presence of serum, growth factors, and tyrosine kinase receptor activation. In this regard, it acts like a dominantly acting oncogene.

Second, its kinase activity is not regulated by posttranslational modifications. Once the protein is expressed, it is active. The regulation of its expression, depending on translation and degradation, determines where and when it will function.

Third, Mos appeared early during animal evolution as a specific oocyte-expressed kinase. A particularity of full-grown oocytes is that transcription of genes is silent: transcriptional activities stop at the end of the growth period and are reinitiated in the cleaving embryos, after fertilization. In this oocyte physiological background, even if the Mos-MAPK pathway would phosphorylate and activate transcription factors, this would not induce transcription of any genes in the oocyte.

From these observations, the apparent conflicting nature of Mos, acting physiologically as a tumour-supressor gene in the unfertilized oocyte, and as an oncogene when inappropriately expressed in somatic cells, seems to be basically resolved: the functions played by Mos depend on the identity of the final MAPK targets that are at its disposal. 

When expressed in somatic cells, MAPK activated by Mos can phosphorylate and stabilize transcription factors as c-Fos and c-Myc, leading to transcriptional induction of critical oncogenes and cellular transformation [135, 136]. Clearly, this cannot happen in oocyte where transcription is inactive. It is also probable that Mos can impose a meiotic-like phenotype on all stages of the somatic cell cycle. In particular, its meiotic regulatory activities concerning the formation of microtubular spindles, the cohesion of sister chromatids and the omission of S-phase, could participate to the chromosome instability characterizing malignant clones where meiotic genes as *mos* are induced [100].

Physiologically, Mos functions in regulating core specializations of female meiosis. Asymmetric spindle positioning and polar body emission as well as cytostatic arrest are the ancestral functions of Mos for Eumetazoa. Interestingly, despite the differences in the meiotic arrest stages of unfertilized oocytes among species, all of them are under the control of Mos, indicating again that the differences rely on particular downstream targets of MAPK. If the translational regulation of Mos is modified, in time or in space, Mos can acquire new roles by finding new targets. This scenario could have operated in the oocyte during evolution, for example, after sequence changes in its UTRs affecting translation timing.

## References

[1] Oskarsson M, McClements WL, Blair DG (1980). Properties of a normal mouse cell DNA sequence (sarc) homologous to the src sequence of Moloney sarcoma virus. *Science*.

[2] Maxwell SA, Arlinghaus RB (1985). Serine kinase activity associated with Moloney murine sarcoma virus-124-encoded p37(mos). *Virology*.

[3] Seth A, Vande Woude GF (1985). Nucleotide sequence and biochemical activities of the Moloney murine sarcoma virus strain HT-1 mos gene. *Journal of Virology*.

[4] Paules RS, Buccione R, Moschel RC, Vande Woude GF, Eppig JJ (1989). Mouse Mos protooncogene product is present and functions during oogenesis. *Proceedings of the National Academy of Sciences of the United States of America*.

[5] Propst F, Rosenberg MP, Iyer A, Kaul K, Vande Woude GF (1987). c-mos Proto-oncogene RNA transcripts in mouse tissues: structural features, developmental regulation, and localization in specific cell types. *Molecular and Cellular Biology*.

[6] Sagata N, Oskarsson M, Copeland T, Brumbaugh J, Woude GFV (1988). Function of c-mos proto-oncogene product in meiotic maturation in Xenopus oocytes. *Nature*.

[7] Watanabe N, Vande Woude GF, Ikawa Y, Sagata N (1989). Specific proteolysis of the c-mos proto-oncogene product by calpain on fertilization of Xenopus eggs. *Nature*.

[8] Tachibana K, Tanaka D, Isobe T, Kishimoto T (2000). c-Mos forces the mitotic cell cycle to undergo meiosis II to produce haploid gametes. *Proceedings of the National Academy of Sciences of the United States of America*.

[9] Amiel A, Leclère L, Robert L, Chevalier S, Houliston E (2009). Conserved functions for Mos in eumetazoan oocyte maturation revealed by studies in a cnidarian. *Current Biology*.

[10] Goldman DS, Kiessling AA, Millette CF, Cooper GM (1987). Expression of c-mos RNA in germ cells of male and female mice. *Proceedings of the National Academy of Sciences of the United States of America*.

[11] Sagata N, Daar I, Oskarsson M, Showalter SD, Vande Woude GF (1989). The product of the mos proto-oncogene as a candidate ’initiator’ for oocyte maturation. *Science*.

[12] Sagata N, Watanabe N, Vande Woude GF, Ikawa Y (1989). The c-mos proto-oncogene product is a cytostatic factor responsible for meiotic arrest in vertebrate eggs. *Nature*.

[13] Hashimoto N, Watanabe N, Furuta Y (1994). Parthenogenetic activation of oocytes in c-mos-deficient mice. *Nature*.

[14] Colledge WH, Carlton MBL, Udy GB, Evans MJ (1994). Disruption of c-mos causes parthenogenetic development of unfertilized mouse eggs. *Nature*.

[15] Hannink M, Donoghue DJ (1985). Lysine residue 121 in the proposed ATP-binding site of the v-mos protein is required for transformation. *Proceedings of the National Academy of Sciences of the United States of America*.

[16] Yew N, Oskarsson M, Daar I, Blair DG, Vande Woude GF (1991). mos gene transforming efficiencies correlate with oocyte maturation and cytostatic factor activities. *Molecular and Cellular Biology*.

[17] Freeman RS, Meyer AN, Li J, Donoghue DJ (1992). Phosphorylation of conserved serine residues does not regulate the ability of mos(xe) protein kinase to induce oocyte maturation or function as cytostatic factor. *Journal of Cell Biology*.

[18] Puls A, Proikas-Cezanne T, Marquardt B, Propst F, Stabel S (1995). Kinase activities of c-Mos and v-Mos proteins: a single amino acid exchange is responsible for constitutive acthation of the 124 v-Mos kinase. *Oncogene*.

[19] Fukasawa K, Zhou R, Matten WT (1995). Mutagenic analysis of functional domains of the mos proto-oncogene and identification of the sites important for MAPK activation and DNA binding. *Oncogene*.

[20] Seth A, Priel E, Vande Woude GF (1987). Nucleoside triphosphate-dependent DNA-binding properties of mos protein. *Proceedings of the National Academy of Sciences of the United States of America*.

[21] Van Beveren C, Van Straaten F, Galleshaw JA, Verma IM (1981). Nucleotide sequence of the genome of a murine sarcoma virus. *Cell*.

[22] Freeman RS, Kanki JP, Ballantyne SM, Pickham KM, Donoghue DJ (1990). Effects of the v-mos oncogene on Xenopus development: meiotic induction in oocytes and mitotic arrest in cleaving embryos. *Journal of Cell Biology*.

[23] Freeman RS, Pickham KM, Kanki JP, Lee BA, Pena SV, Donoghue DJ (1989). Xenopus homolog of the mos protooncogene transforms mammalian fibroblasts and induces maturation of Xenopus oocytes. *Proceedings of the National Academy of Sciences of the United States of America*.

[24] Freeman RS, Donoghue DJ (1989). Transforming mutant v-mos protein kinases that are deficient in in vitro autophosphorylation. *Molecular and Cellular Biology*.

[25] Posada J, Yew N, Ahn NG, Vande Woude GF, Cooper JA (1993). Mos stimulates MAP kinase in Xenopus oocytes and activates a MAP kinase kinase in vitro. *Molecular and Cellular Biology*.

[26] Shibuya EK, Ruderman JV (1993). Mos induces the in vitro activation of mitogen-activated protein kinases in lysates of frog oocytes and mammalian somatic cells. *Molecular Biology of the Cell*.

[27] Nebreda AR, Hill C, Gomez N, Cohen P, Hunt T (1993). The protein kinase mos activates MAP kinase kinase in vitro and stimulates the MAP kinase pathway in mammalian somatic cells in vivo. *FEBS Letters*.

[28] Nebreda AR, Hunt T (1993). The c-mos proto-oncogene protein kinase turns on and maintains the activity of MAP kinase, but not MPF, in cell-free extracts of Xenopus oocytes and eggs. *EMBO Journal*.

[29] Resing KA, Mansour SJ, Hermann AS (1995). Determination of v-Mos-catalyzed phosphorylation sites and autophosphorylation sites on MAP kinase kinase by ESI/MS. *Biochemistry*.

[30] Pham CD, Arlinghaus RB, Zheng CF, Guan KL, Singh B (1995). Characterization of MEK1 phosphorylation by the v-Mos protein. *Oncogene*.

[31] Ferrell JE, Machleder EM (1998). The biochemical basis of an all-or-none cell fate switch in xenopus oocytes. *Science*.

[32] Ferrell JE, Wu M, Gerhart JC, Martin GS (1991). Cell cycle tyrosine phosphorylation of p34(cdc2) and a microtubule-associated protein kinase homolog in Xenopus oocytes and eggs. *Molecular and Cellular Biology*.

[33] Nishizawa M, Okazaki K, Furuno N, Watanabe N, Sagata N (1992). The ’second-codon rule’ and autophosphorylation govern the stability and activity of Mos during the meiotic cell cycle in Xenopus oocytes. *EMBO Journal*.

[34] Roy LM, Singh B, Gautier J, Arlinghaus RB, Nordeen SK, Maller JL (1990). The cyclin B2 component of MPF is a substrate for the c-mos(xe) proto-oncogene product. *Cell*.

[35] Zhou RP, Shen RL, Pinto da Silva P, Vande Woude GF (1991). In vitro and in vivo characterization of pp39mos association with tubulin. *Cell Growth & Differentiation*.

[36] Zhou R, Oskarsson M, Paules RS, Schulz N, Cleveland D, Vande Woude GF (1991). Ability of the c-mos product to associate with and phosphorylate tubulin. *Science*.

[37] Bai W, Singh B, Yang Y (1992). The physical interaction between p37env-mos and tubulin structures. *Oncogene*.

[38] Verlhac MH, Kubiak JZ, Weber M (1996). Mos is required for MAP kinase activation and is involved in microtubule organization during meiotic maturation in the mouse. *Development*.

[39] Bodart JFL, Baert FY, Sellier C, Duesbery NS, Flament S, Vilain JP (2005). Differential roles of p39-Xp42 cascade proteins on Raf1 phosphorylation and spindle morphogenesis in Xenopus oocytes. *Developmental Biology*.

[40] Verlhac MH, Lefebvre C, Kubiak JZ (2000). Mos activates MAP kinase in mouse oocytes through two opposite pathways. *EMBO Journal*.

[41] Ivanovska I, Lee E, Kwan KM, Fenger DD, Orr-Weaver TL (2004). The Drosophila MOS ortholog is not essential for meiosis. *Current Biology*.

[42] Yamamoto TG, Watanabe S, Essex A, Kitagawa R (2008). Spdl-1 functions as a kinetochore receptor for MDF-1 in Caenorhabditis elegans. *Journal of Cell Biology*.

[43] Masui Y, Markert CL (1971). Cytoplasmic control of nuclear behavior during meiotic maturation of frog oocytes. *Journal of Experimental Zoology*.

[44] Ciemerych MA, Kubiak JZ (1998). Cytostatic activity develops during meiosis I in oocytes of LT/Sv mice. *Developmental Biology*.

[45] Wilkie GS, Dickson KS, Gray NK (2003). Regulation of mRNA translation by 5′- and 3′-UTR-binding factors. *Trends in Biochemical Sciences*.

[46] De Moor CH, Meijer H, Lissenden S (2005). Mechanisms of translational control by the 3′ UTR in development and differentiation. *Seminars in Cell and Developmental Biology*.

[47] Schwab MS, Kim SH, Terada N (1999). p70(S6K) controls selective mRNA translation during oocyte maturation and early embryogenesis in Xenopus laevis. *Molecular and Cellular Biology*.

[48] Sheets MD, Wu M, Wickens M (1995). Polyadenylation of c-mos mRNA as it control point in Xenopus meiotic maturation. *Nature*.

[49] Kuge H, Brownlee GG, Gershon PD, Richter JD (1998). Cap ribose methylation of c-mos mRNA stimulates translation and oocyte maturation in Xenopus laevis. *Nucleic Acids Research*.

[50] Hake LE, Richter JD (1994). CPEB is a specificity factor that mediates cytoplasmic polyadenylation during Xenopus oocyte maturation. *Cell*.

[51] Stebbins-Boaz B, Cao Q, De Moor CH, Mendez R, Richter JD (1999). Maskin is a CPEB-associated factor that transiently interacts with eIF-4E. *Molecular Cell*.

[52] Stebbins-Boaz B, Hake LE, Richter JD (1996). CPEB controls the cytoplasmic polyadenylation of cyclin, Cdk2 and c-mos mRNAs and is necessary for oocyte maturation in Xenopus. *EMBO Journal*.

[53] Mendez R, Hake LE, Andresson T, Littlepage LE, Ruderman JV, Richter JD (2000). Phosphorylation of CPE binding factor by Eg2 regulates translation of c-mos mRNA. *Nature*.

[54] Barnard DC, Ryan K, Manley JL, Richter JD (2004). Symplekin and xGLD-2 are required for CPEB-mediated cytoplasmic polyadenylation. *Cell*.

[55] Charlesworth A, Ridge JA, King LA, MacNicol MC, MacNicol AM (2002). A novel regulatory element determines the timing of Mos mRNA translation during Xenopus oocyte maturation. *EMBO Journal*.

[56] Charlesworth A, Cox LL, MacNicol AM (2004). Cytoplasmic polyadenylation element (CPE)- and CPE-binding protein (CPEB)-independent mechanisms regulate early class maternal mRNA translational activation in Xenopus oocytes. *Journal of Biological Chemistry*.

[57] Charlesworth A, Wilczynska A, Thampi P, Cox LL, MacNicol AM (2006). Musashi regulates the temporal order of mRNA translation during Xenopus oocyte maturation. *EMBO Journal*.

[58] Arumugam K, Wang Y, Hardy LL, MacNicol MC, MacNicol AM (2010). Enforcing temporal control of maternal mRNA translation during oocyte cell-cycle progression. *EMBO Journal*.

[59] Prasad CK, Mahadevan M, MacNicol MC, MacNicol AM (2008). Mos 3′ UTR regulatory differences underlie species-specific temporal patterns of Mos mRNA cytoplasmic polyadenylation and translational recruitment during oocyte maturation. *Molecular Reproduction and Development*.

[60] Choi T, Rulong S, Resau J (1996). Mos/mitogen-activated protein kinase can induce early meiotic phenotypes in the absence of maturation-promoting factor: a novel system for analyzing spindle formation during meiosis I. *Proceedings of the National Academy of Sciences of the United States of America*.

[61] Gotoh Y, Masuyama N, Dell K, Shirakabe K, Nishida E (1995). Initiation of Xenopus oocyte maturation by activation of the mitogen- activated protein kinase cascade. *Journal of Biological Chemistry*.

[62] Howard EL, Charlesworth A, Welk J, MacNicol AM (1999). The mitogen-activated protein kinase signaling pathway stimulates Mos mRNA cytoplasmic polyadenylation during Xenopus oocyte maturation. *Molecular and Cellular Biology*.

[63] Keady BT, Kuo P, Martínez SE, Yuan L, Hake LE (2007). MAPK interacts with XGef and is required for CPEB activation during meiosis in Xenopus oocytes. *Journal of Cell Science*.

[64] Verlhac MH, De Pennart H, Maro B, Cobb MH, Clarke HJ (1993). MAP kinase becomes stably activated at metaphase and is associated with microtubule-organizing centers during meiotic maturation of mouse oocytes. *Developmental Biology*.

[65] Tan X, Chen DY, Yang Z (2001). Phosphorylation of p90 during meiotic maturation and parthenogenetic activation of rat oocytes: Correlation with MAP kinases. *Zygote*.

[66] Gebauer F, Xu W, Cooper GM, Richter JD (1994). Translational control by cytoplasmic polyadenylation of c-mos mRNA is necessary for oocyte maturation in the mouse. *EMBO Journal*.

[67] Gebauer F, Richter JD (1996). Mouse cytoplasmic polyadenylylation element binding protein: an evolutionarily conserved protein that interacts with the cytoplasmic polyadenylylation elements of c-mos mRNA. *Proceedings of the National Academy of Sciences of the United States of America*.

[68] Hodgman R, Tay J, Mendez R, Richter JD (2001). CPEB phosphorylation and cytoplasmic polyadenylation are catalyzed by the kinase IAK1/Eg2 in maturing mouse oocytes. *Development*.

[69] Racki WJ, Richter JD (2006). CPEB controls oocyte growth and follicle development in the mouse. *Development*.

[70] Belloc E, Méndez R (2008). A deadenylation negative feedback mechanism governs meiotic metaphase arrest. *Nature*.

[71] Belloc E, Piqué M, Méndez R (2008). Sequential waves of polyadenylation and deadenylation define a translation circuit that drives meiotic progression. *Biochemical Society Transactions*.

[72] Nebreda AR, Gannon JV, Hunt T (1995). Newly synthesized protein(s) must associate with p34(cdc2) to activate MAP kinase and MPF during progesterone-induced maturation of Xenopus oocytes. *EMBO Journal*.

[73] Frank-Vaillant M, Jessus C, Ozon R, Maller JL, Haccard O (1999). Two distinct mechanisms control the accumulation of cyclin B1 and Mos in Xenopus oocytes in response to progesterone. *Molecular Biology of the Cell*.

[74] Nishizawa M, Furuno N, Okazaki K, Tanaka H, Ogawa Y, Sagata N (1993). Degradation of Mos by the N-terminal proline (Pro)-dependent ubiquitin pathway on fertilization of Xenopus eggs: possible significance of natural selection for Pro in Mos. *EMBO Journal*.

[75] Castro A, Peter M, Magnaghi-Jaulin L (2001). Cyclin B/cdc2 induces c-Mos stability by direct phosphorylation in Xenopus oocytes. *Molecular Biology of the Cell*.

[76] Pham CD, Vuyyuru VB, Yang Y, Bai W, Singh B (1999). Evidence for an important role of serine 16 and its phosphorylation in the stabilization of c-Mos. *Oncogene*.

[77] Weber M, Kubiak JZ, Arlinghaus RB, Pines J, Maro B (1991). c-mos Proto-oncogene product is partly degraded after release from meiotic arrest and persists during interphase in mouse zygotes. *Developmental Biology*.

[78] Phillips KP, Petrunewich MAF, Collins JL, Booth RA, Liu XJ, Baltz JM (2002). Inhibition of MEK or cdc2 kinase parthenogenetically activates mouse eggs and yields the same phenotypes as Mosparthenogenotes. *Developmental Biology*.

[79] Lorca T, Galas S, Fesquet D, Devault A, Cavadore JC, Doree M (1991). Degradation of the proto-oncogene product p39(mos) is not necessary for cyclin proteolysis and exit from meiotic metaphase: requirement for a Ca-calmodulin dependent event. *EMBO Journal*.

[80] Wasserman WJ, Masui Y (1975). Effects of cycloheximide on a cytoplasmic factor initiating meiotic maturation in Xenopus oocytes. *Experimental Cell Research*.

[81] Yew N, Mellini ML, Vande Woude GF (1992). Meiotic initiation by the mos protein in Xenopus. *Nature*.

[82] Haccard O, Lewellyn A, Hartley RS, Erikson E, Maller JL (1995). Induction of Xenopus oocyte meiotic maturation by MAP kinase. *Developmental Biology*.

[83] Huang W, Kessler DS, Erikson RL (1995). Biochemical and biological analysis of Mek1 phosphorylation site mutants. *Molecular Biology of the Cell*.

[84] Gross SD, Lewellyn AL, Maller JL (2001). A constitutively active form of the protein kinase p90Rsk1 is sufficient to trigger the G2/M transition in Xenopus oocytes. *Journal of Biological Chemistry*.

[85] Gross SD, Schwab MS, Taieb FE, Lewellyn AL, Qian YW, Maller JL (2000). The critical role of the MAP kinase pathway in meiosis II in Xenopus oocytes is mediated by p90(Rsk). *Current Biology*.

[86] Palmer A, Gavin AC, Nebreda AR (1998). A link between MAP kinase and p34(cdc2)/cyclin B during oocyte maturation: p90(rsk) phosphorylates and inactivates the p34(cdc2) inhibitory kinase Myt1. *EMBO Journal*.

[87] Peter M, Labbé JC, Dorée M, Mandart E (2002). A new role for Mos in Xenopus oocyte maturation: targeting Myt1 independently of MAPK. *Development*.

[88] Haccard O, Jessus C (2006). Oocyte maturation, Mos and cyclins—a matter of synthesis: two functionally redundant ways to induce meiotic maturation. *Cell Cycle*.

[89] Fisher DL, Brassac T, Galas S, Dorée M (1999). Dissociation of MAP kinase activation and MPF activation in hormone-stimulated maturation of Xenopus oocytes. *Development*.

[90] Dupré A, Jessus C, Ozon R, Haccard O (2002). Mos is not required for the initiation of meiotic maturation in Xenopus oocytes. *EMBO Journal*.

[91] Lemaître JM, Bocquet S, Méchali M (2002). Competence to replicate in the unfertilized egg is conferred by Cdc6 during meiotic maturation. *Nature*.

[92] Whitmire E, Khan B, Coué M (2002). Cdc6 synthesis regulates replication competence in Xenopus oocytes. *Nature*.

[93] Furuno N, Nishizawa M, Okazaki K (1994). Suppression of DNA replication via mos function during meiotic divisions in xenopus oocytes. *EMBO Journal*.

[94] O’Keefe SJ, Wolfes H, Kiessling AA, Cooper GM (1989). Microinjection of antisense c-mos oligonucleotides prevents meiosis II in the maturing mouse egg. *Proceedings of the National Academy of Sciences of the United States of America*.

[95] O’Keefe SJ, Kiessling AA, Cooper GM (1991). The c-mos gene product is required for cyclin B accumulation during meiosis of mouse eggs. *Proceedings of the National Academy of Sciences of the United States of America*.

[96] Hochegger H, Klotzbücher A, Kirk J (2001). New B-type cyclin synthesis is required between meiosis I and II during Xenopus oocyte maturation. *Development*.

[97] Tong C, Fan HY, Chen DY, Song XF, Schatten H, Sun QY (2003). Effects of MEK inhibitor U0126 on meiotic progression in mouse oocytes: microtuble organization, asymmetric division and metaphase II arrest. *Cell Research*.

[98] Araki K, Naito K, Haraguchi S (1996). Meiotic abnormalities of c-mos knockout mouse oocytes: activation after first meiosis or entrance into third meiotic metaphase. *Biology of Reproduction*.

[99] Yu LZ, Xiong B, Gao WX (2007). MEK1/2 regulates microtubule organization, spindle pole tethering and asymmetric division during mouse oocyte meiotic maturation. *Cell Cycle*.

[100] Erenpreisa J, Cragg MS (2010). MOS, aneuploidy and the ploidy cycle of cancer cells. *Oncogene*.

[101] Haccard O, Sarcevic B, Lewellyn A (1993). Induction of metaphase arrest in cleaving xenopus embryos by MAP kinase. *Science*.

[102] Gross SD, Schwab MS, Lewellyn AL, Maller JL (1999). Induction of metaphase arrest in cleaving Xenopus embryos by the protein kinase p90(Rsk). *Science*.

[103] Bhatt RR, Ferrell JE (1999). The protein kinase p90 Rsk as an essential mediator of cytostatic factor activity. *Science*.

[104] Kosako H, Gotoh Y, Nishida E (1994). Mitogen-activated protein kinase kinase is required for the Mos-induced metaphase arrest. *Journal of Biological Chemistry*.

[105] Abrieu A, Lorca T, Labbé JC, Morin N, Keyse S, Dorée M (1996). MAP kinase does not inactivate, but rather prevents the cyclin degradation pathway from being turned on in Xenopus egg extracts. *Journal of Cell Science*.

[106] Daar I, Paules RS, Vande Woude GF (1991). A characterization of cytostatic factor activity from Xenopus eggs and c-mos-transformed cells. *Journal of Cell Biology*.

[107] MacNicol AM, Muslin AJ, Howard EL, Kikuchi A, MacNicol MC, Williams LT (1995). Regulation of Raf-1-dependent signaling during early Xenopus development. *Molecular and Cellular Biology*.

[108] Lorca T, Cruzalegui FH, Fesquet D (1993). Calmodulin-dependent protein kinase II mediates inactivation of MPF and CSF upon fertilization of Xenopus eggs. *Nature*.

[109] Murray AW (2004). Recycling the cell cycle: cyclins revisited. *Cell*.

[110] Liu J, Maller JL (2005). Calcium elevation at fertilization coordinates phosphorylation of XErp1/Emi2 by Plx1 and CaMK II to release metaphase arrest by cytostatic factor. *Current Biology*.

[111] Liu J, Grimison B, Lewellyn AL, Maller JL (2006). The anaphase-promoting complex/cyclosome inhibitor Emi2 Is essential for meiotic but not mitotic cell cycles. *Journal of Biological Chemistry*.

[112] Tang W, Wu JQ, Guo Y (2008). Cdc2 and Mos regulate Emi2 stability to promote the meiosis I-meiosis II transition. *Molecular Biology of the Cell*.

[113] Rauh NR, Schmidt A, Bormann J, Nigg EA, Mayer TU (2005). Calcium triggers exit from meiosis II by targeting the APC/C inhibitor XErp1 for degradation. *Nature*.

[114] Schmidt A, Duncan PI, Rauh NR (2005). Xenopus polo-like kinase Plx1 regulates XErp1, a novel inhibitor of APC/C activity. *Genes and Development*.

[115] Inoue D, Ohe M, Kanemori Y, Nobui T, Sagata N (2007). A direct link of the Mos-MAPK pathway to Erp1/Emi2 in meiotic arrest of Xenopus laevis eggs. *Nature*.

[116] Nishiyama T, Ohsumi K, Kishimoto T (2007). Phosphorylation of Erp1 by p90rsk is required for cytostatic factor arrest in Xenopus laevis eggs. *Nature*.

[117] Wu JQ, Hansen DV, Guo Y (2007). Control of Emi2 activity and stability through Mos-mediated recruitment of PP2A. *Proceedings of the National Academy of Sciences of the United States of America*.

[118] Wu Q, Guo Y, Yamada A (2007). A role for Cdc2- and PP2A-mediated regulation of Emi2 in the maintenance of CSF arrest. *Current Biology*.

[119] Shoji S, Yoshida N, Amanai M (2006). Mammalian Emi2 mediates cytostatic arrest and transduces the signal for meiotic exit via Cdc20. *EMBO Journal*.

[120] Madgwick S, Hansen DV, Levasseur M, Jackson PK, Jones KT (2006). Mouse Emi2 is required to enter meiosis II by reestablishing cyclin B1 during interkinesis. *Journal of Cell Biology*.

[121] Kalab P, Kubiak JZ, Verlhac MH, Colledge WH, Maro B (1996). Activation of p90 during meiotic maturation and first mitosis in mouse oocytes and eggs: MAP kinase-independent and -dependent activation. *Development*.

[122] Dumont J, Umbhauer M, Rassinier P, Hanauer A, Verlhac MH (2005). p90Rsk is not involved in cytostatic factor arrest in mouse oocytes. *Journal of Cell Biology*.

[123] Mori M, Hara M, Tachibana K, Kishimoto T (2006). p90 is required for G1 phase arrest in unfertilized starfish eggs. *Development*.

[124] Tachibana K, Mori M, Matsuhira T (2010). Initiation of DNA replication after fertilization is regulated by p90Rsk at pre-RC/pre-IC transition in starfish eggs. *Proceedings of the National Academy of Sciences of the United States of America*.

[125] Hara M, Mori M, Wada T, Tachibana K, Kishimoto T (2009). Start of the embryonic cell cycle is dually locked in unfertilized starfish eggs. *Development*.

[126] McKim KS, Jang JK, Theurkauf WE, Hawley RS (1993). Mechanical basis of meiotic metaphase arrest. *Nature*.

[127] Jang JK, Messina L, Erdman MB, Arbel T, Hawley RS (1995). Induction of metaphase arrest in Drosophila oocytes by chiasma-based kinetochore tension. *Science*.

[128] Yamamoto DS, Tachibana K, Sumitani M, Lee JM, Hatakeyama M (2008). Involvement of Mos-MEK-MAPK pathway in cytostatic factor (CSF) arrest in eggs of the parthenogenetic insect, Athalia rosae. *Mechanisms of Development*.

[129] Russo GL, Bilotto S, Ciarcia G, Tosti E (2009). Phylogenetic conservation of cytostatic factor related genes in the ascidian Ciona intestinalis. *Gene*.

[130] Marino M, Wilding M, Dale B (2000). Interaction of cell cycle kinases, microtubules, and chromatin in ascidian oocytes during meiosis. *Molecular Reproduction and Development*.

[131] Church DL, Guan KL, Lambie EJ (1995). Three genes of the MAP kinase cascade, mek-2, mpk-1/sur-1 and let-60 ras, are required for meiotic cell cycle progression in Caenorhabditis elegans. *Development*.

[132] Page BD, Guedes S, Waring D, Priess JR (2001). The C. elegans E2F- and DP-related proteins are required for embryonic asymmetry and negatively regulate Ras/MAPK signaling. *Molecular Cell*.

[133] Miller MA, Nguyen VQ, Lee MH (2001). A sperm cytoskeletal protein that signals oocyte meiotic maturation and ovulation. *Science*.

[134] Hajnal A, Berset T (2002). The C. elegans MAPK phosphatase LIP-1 is required for the G/M meiotic arrest of developing oocytes. *EMBO Journal*.

[135] Davis RJ (1993). The mitogen-activated protein kinase signal transduction pathway. *Journal of Biological Chemistry*.

[136] Hill CS, Marais R, John S, Wynne J, Dalton S, Treisman R (1993). Functional analysis of a growth factor-responsive transcription factor complex. *Cell*.

